# Elevated Soluble CD163 in Gestational Diabetes Mellitus: Secretion from Human Placenta and Adipose Tissue

**DOI:** 10.1371/journal.pone.0101327

**Published:** 2014-07-01

**Authors:** Muhammad Furqan Bari, Martin O. Weickert, Kavitha Sivakumar, Sean G. James, David R. J. Snead, Bee Kang Tan, Harpal Singh Randeva, Claire Cecile Bastie, Manu Vatish

**Affiliations:** 1 Department of Pathology, Dow International Medical College, Karachi, Pakistan; 2 Divisions of Reproduction and Metabolic & Vascular Health, Warwick Medical School, Coventry, West Midlands, United Kingdom; 3 Departments of Pathology & Endocrinology, University Hospitals Coventry and Warwickshire NHS Trust, Coventry, West Midlands, United Kingdom; 4 Department of Medicine & Endocrinology, Albert Einstein College of Medicine, Bronx, New York, United States of America; 5 Nuffield Department of Obstetrics & Gynaecology, University of Oxford, Oxford, Oxfordshire, United Kingdom; Xavier Bichat Medical School, INSERM-CNRS - Université Paris Diderot, France

## Abstract

Recently soluble CD163 (sCD163), a cleaved form of the macrophage receptor CD163, was identified as a macrophage-specific risk-predictor for developing Type 2 Diabetes. Here, we investigate circulating levels of sCD163 in gestational diabetes mellitus (GDM). Furthermore, given the role of the placenta in the pathogenesis of GDM, we assessed placental contribution to sCD163 secretion. Paired maternal (venous) and umbilical vein blood samples from GDM (n = 18) and Body Mass Index (BMI) matched control women (n = 20) delivered by caesarean section at 39–40 week gestation were assessed for circulating levels of sCD163, Tumour necrosis factor alpha (TNF-α) and Interleukin 6 (IL-6). Media from explant culture of maternal subcutaneous fat and corresponding placental tissues were assayed for these same molecules. CD163 positive cell numbers were determined in placental and adipose tissues of GDM and control women. We found significantly elevated circulating sCD163 levels in GDM mothers (688.4±46.9 ng/ml vs. 505.6±38.6 ng/ml) and their offspring (418.2±26.6 ng/ml vs. 336.3±24.4 ng/ml [p<0.05 for both]) as compared to controls, together with elevated circulating TNF-α and IL-6 levels. Moreover, both GDM placentae (268.1±10.8 ng/ml/mg vs. 187.6±20.6 ng/ml/mg) and adipose explants (41.1±2.7 ng/ml/mg vs. 26.6±2.4 ng/ml/mg) released significantly more sCD163 than controls. Lastly, significantly more CD163 positive cells were observed in GDM placentae (25.7±1.1 vs. 22.1±1.2) and adipose tissue (19.1±1.1 vs 12.7±0.9) compared to controls. We describe elevated sCD163 levels in GDM and identify human placenta as a novel source of sCD163 suggesting that placental tissues might contribute to the increased levels of circulating sCD163 in GDM pregnancies.

## Introduction

Gestational diabetes mellitus (GDM) is defined as any degree of glucose intolerance with onset or first recognition during pregnancy[1] and is the most common metabolic complication of pregnancy [2], with an increasing prevalence directly linked to increasing obesity[3]. GDM is a risk factor for developing T2DM later in life [4], with up to 70% of women with GDM developing this disorder within the first 5 years following their pregnancy [5]. Moreover, the offspring of women with GDM are also at increased lifetime risk of obesity and T2DM [6]. Intriguingly, GDM is linked to placental function and a number of placenta-derived molecules have been implicated in causing the insulin resistance in GDM [7]. However, since many of the placenta-secreted molecules are also produced by adipose tissue, it is difficult to segregate the respective roles of adipose tissue and placenta in the insulin resistance of GDM. Chronic inflammation in adipose tissue plays an important role in the development of insulin resistance particularly in obesity [8]. This inflammation is associated with macrophage infiltration into adipose tissue and macrophage-derived secretion of pro-inflammatory cytokines (e.g. TNF-α, IL-6), which promote insulin resistance and might have direct implications in obesity related disease, particularly Type 2 Diabetes (T2DM) [9], [10].

Recently, soluble CD163 (sCD163) was identified as a macrophage-specific risk-predictor for developing T2DM [11]. sCD163 is derived from the cleavage of membrane-bound CD163, a macrophage receptor involved in scavenging haptoglobin-haemoglobin complexes [12]. Shedding of CD163 occurs physiologically, with detectable levels found in normal individuals [13] and in pregnancy[14]. Parkner et al [15] hypothesized that circulating sCD163 levels are linked to CD163 expression and macrophage content in adipose tissue. Consistent with this, concentrations of sCD163 are increased in obesity[16] and T2DM [15], [17]. There is recent evidence that sCD163 is specifically associated with CD163 mRNA expression and insulin sensitivity in adipose tissue [18].

Despite the fact that sCD163 levels are increased in T2DM [11], levels of sCD163 in GDM have not been explored. In this study, we therefore analysed the circulating levels of sCD163 together with known pro-inflammatory markers (TNF-α and IL-6) in pregnancies complicated by GDM, and compared these markers to BMI matched controls without GDM. Importantly, the placental macrophage population, known as Hofbauer cells (HBC), is also characterized by the presence of CD163 as a surface marker [19], [20], suggesting that the placenta might participate in the secretion of sCD163. We therefore further explore whether the human placenta might represent a source of sCD163.

## Materials and Methods

### Subjects

All participants were pregnant Caucasian women scheduled for elective caesarean section delivering at 39–40 weeks of gestation, for the sampling of fasted blood from midnight prior to surgery. Eighteen GDM [BMI >30 (mean age 34.75±1.71 years)] and 20 BMI-matched controls [BMI >30 (mean age 33.21±1.02 years)]. The indications for surgery were breech presentation or previous Caesarean section. Procedures were performed between 0830 and 1230 for all individuals. The National Research Ethics Service (Coventry Local Research Ethics Committee) approved this study and all patients gave written informed consent (Research Ethics Committees 07/H1210/141) in accordance with the Declaration of Helsinki. Oral glucose tolerance tests (OGTTs) were performed at 26–28 weeks of gestation in all participants to diagnose gestational diabetes. Criteria for a diagnosis of GDM were made according to the cut-off values established by guidelines from the National Institute of Clinical Excellence [21].

Women with multiple pregnancies, as well as patients with cardiovascular diseases, diabetes, pre-eclampsia and/or other diseases were excluded. Paired maternal venous and umbilical cord venous blood samples were collected at the time of caesarean section and were spun (3,000×g -Beckman Coulter DS-9623C) immediately. Supernatants were immediately stored at −80°C. All chemicals and reagents were from Sigma-Aldrich (Gillingham, UK) unless otherwise stated.

### Biochemical & Hormone Analysis

Venous blood samples were collected without anticoagulants for the measurement of sCD163, insulin, glucose and for lipid profiling. Plasma glucose levels quantification and lipid profile analysis were performed on a Roche modular automated chemical analyser (Roche Diagnostics Scandinavian, Bromma, Sweden). Insulin concentrations were measured using a human Insulin ELISA kit from Invitrogen (Camarillo, CA, USA). The score of insulin resistance by homeostasis model assessment (HOMA-IR) was calculated as described previously [22]. Non-esterified free fatty acids (NEFA) were measured using a kit from BioVision Research Products (Milpitas, CA, USA). Plasma sCD163 concentrations were determined using a human ELISA kit (R & D Quantikine, Abingdon, UK). The minimum detection limit for the assay was 0.058 ng/ml. The intra-assay coefficient of variation was ≤3.8% and inter-assay coefficient of variation was ≤6.7%. Serum leptin was measured using a human ELISA kit (R & D Quantikine, Abingdon, UK). The minimum detection limit for the assay was 7.8 pg/ml. The intra-assay coefficient of variation was 3.2% and inter-assay coefficient of variation was 4.2%. All measurements were performed in triplicate.

### Tissue explant culture

Maternal subcutaneous abdominal fat explants and corresponding placental explants from GDM and control (n = 4 for both) patients were prepared as previously described [23], [24]. Briefly, areas of each placenta were randomly sampled and villous tissue was cultured on Costar Netwell plates (Corning B.V. Amsterdam, Netherlands). Adipose tissue explants were cultured in 6-well plates. Explants were cultured in CMRL-1066 medium (Invitrogen, Paisley, UK) supplemented with 5% Fetal Calf Serum (Invitrogen, Paisley, UK), 100 µg/ml streptomycin sulphate and 100 IU/ml penicillin. Media aliquots were collected after 24 and 48 hrs of incubation, centrifuged at 1,000 rpm (46×*g*) for 5 min and supernatants were stored in −80°C.

### Immunohistochemistry (IHC) and macrophage cell counting

Frozen placental and adipose tissue sections (8 µm, respectively) were obtained using a cryostat (Leica Microsystems, Milton Keynes, UK) and fixed in 100% cold acetone for 10 min. Immunohistochemical analyses were performed on serial sections of placenta and adipose tissues as described previously [25]. Immunostaining for CD163 (Abcam, Cambridge, UK) for placental and adipose tissue macrophages was performed manually at 1∶1000 in 1% BSA in PBST using Leica Polymer detection system (Leica Microsystems) following the manufacturer's recommendations except for the post primary block which was not used. CD163 positive cells, in ten random areas at 40× magnification for placenta and adipose tissues respectively were counted using image J [26] by two authors independently (MFB, SJ) who were blinded to the diagnosis.

### Statistical analyses

Data are expressed as mean ± standard error of the mean (SEM). Differences between two groups were compared using the Mann-Whitney U test, with significance set at a *p* value <0.05. Pearson correlations were used to examine the relationship between variables. Partial intra-class correlations were additionally calculated between sCD163 as the dependent variable and other pro-inflammatory markers, HOMA-IR, and blood lipids as the independent variables, and with simultaneous adjustment for above markers, age, sex and BMI. Stepwise linear regression was performed with presence or absence of GDM as the independent variable, and age, BMI, HOMA-IR, blood lipids, IL-6, TNF-α and sCD163 as the dependent variables.

Analyses were performed using SPSS (IBM, version 21, Amrok, USA). Insulin resistance was estimated using homeostasis model assessment of insulin resistance (HOMA-IR), calculated as [fasting insulin (µIU/mL) x fasting glucose (mmol/L)/22.5][22].

## Results

### Patient characteristics and biochemical profiles

Table 1 shows baseline clinical and biochemical characteristics for the GDM and control groups. As apparent in Table 1, the diagnosis of GDM was confirmed following a 75 g oral glucose tolerance test (OGTT), which had been performed earlier in pregnancy (26–28 weeks gestation). At delivery there were significant differences in lipid parameters in GDM mothers (Table 1). Leptin levels, which correlate with adiposity, were similar in both GDM and control mothers as were gestational weight gain during the pregnancy and thus HOMA-IR was not significantly different between GDM and BMI matched controls, in agreement with previous studies [27]. Similarly, no significant differences were seen in leptin or HOMA-IR values in the fetal cohort.

**Table 1:** Clinical and Biochemical characteristics of patients.

Patient Characteristics	GDM (Mean ± SEM/Range) n = 18	Controls (Mean ± SEM/Range) n = 20	Significance
Maternal Age (yrs)	34.4 (26–47)	33.2 (25–40)	NS
Booking BMI (Kg/m2)	31.2±1.4	31±1.5	NS
Gestational Weight Gain (Kg)	11.5±4.5	10.7±5.1	NS
Mean Gestational Age at Delivery (wks + days)	39+2	39+2	NS
OGTT (2 h) 26–28 wks	8.87±0.25	5.37±0.28	p<0.05*
Birth weight (Kg)	3.45±0.13	33.65±0.06	NS

Biochemical Parameters	Maternal			Cord		
	GDM) (n = 18)	Controls (n = 20)	p value	GDM (n = 18)	Controls (n = 20)	p value
HOMA-IR	4.04±0.97	3.93±0.57	NS	2.16± 0.37	2.19± 0.77	NS
Triglycerides (mmol/L)	3.48±0.33	2.71±0.17	<0.05*	0.25±0.02	0.22±0.02	NS
Total Cholesterol (mmol/L)	6.07±0.31	7.14±0.33	<0.05*	1.72±0.13	1.81±0.12	NS
HDL Cholesterol (mmol/L)	1.28±0.1	1.74±0.07	<0.05*	0.72±0.07	0.82±0.08	NS
LDL Cholesterol (mmol/L)	3.14±0.28	4.15±0.21	<0.05*	1.05±0.14	0.87±0.07	NS
Leptin (ng/mL)	47.61±5.10	47.53±5.34	NS	4.20±0.27	6.92±1.28	NS

**Upper Panel**: Clinical characteristics of GDM and controls utilized in this study. (OGTT =  Oral glucose tolerance test, NS =  Non-significant, p<0.05*  =  statistically significant).

**Lower Panel**: Biochemical profiles of maternal and cord blood from GDM and controls at delivery. (HDL =  High density lipoproteins, LDL =  Low density lipoproteins, HOMA-IR =  homeostasis model assessment of insulin resistance, p<0.05* =  statistically significant.).

* = statistically significant.

### Increased inflammatory markers and sCD163 levels in GDM pregnancy

**Figure 1:**
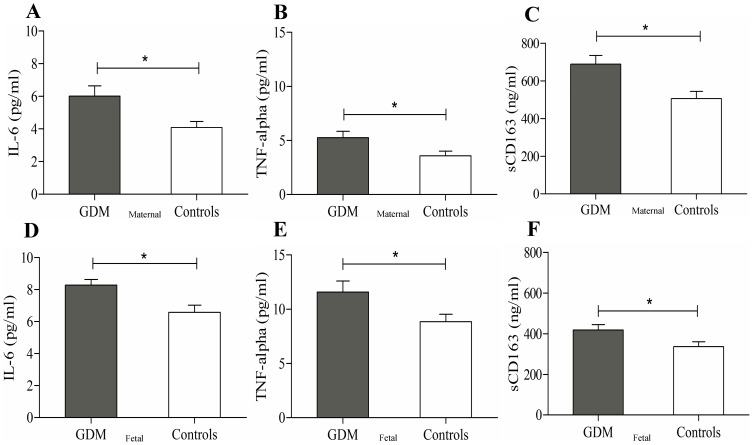
Circulating levels of maternal and fetal IL-6, TNF-alpha and sCD163. ELISA measurements of maternal (A, B and C) and fetal (D, E and F) circulating levels of IL6 (A and D), TNF-alpha (B and E) and sCD163 (C and F). GDM = gestational diabetes; sCD163 = Soluble CD163; IL-6 = Interleukin 6; TNF-alpha = Tumor necrosis factor alpha [*p<0.05 =  statistically significant]) Details in Materials & Methods.

To investigate the inflammatory status of GDM mothers and their offspring, markers of inflammation were first investigated. Consistent with previous reports [28], plasma levels of IL-6 and TNF-α were significantly increased in GDM mothers compared to their non-diabetic controls (6.01±0.6 vs. 4.08±0.4 pg/ml, p<0.01 and 5.25±0.6 pg/ml vs. 3.57±0.4 pg/ml, p<0.03, respectively) (Figures 1A & 1B). Moreover, these pro-inflammatory cytokines were also increased in the offspring of GDM mothers (Figures 1D & 1E). We next determined circulating levels of sCD163 in mothers and their offspring. GDM mothers and their offspring presented significantly elevated levels of sCD163 (688.4±47 ng/ml vs. 505.63±38.6 ng/ml, p<0.005 and 418.20±26.6 ng/ml vs. 336.32±24.4 ng/ml, p<0.003, respectively) (Figures 1C & 1F).

Finally, correlation analyses revealed that when analysing the entire cohort, significant positive correlations were observed between maternal sCD163, maternal IL-6 and fetal IL-6, but not with TNF-α (Table 2). These significant correlations were clearly related to GDM status. Despite the lower number of cases when restricting to the GDM subgroup (n = 18), the observed correlations of sCD163 with IL-6 strongly increased in GDM; but disappeared in the matched controls (Table 2).

**Table 2:** Correlation Analyses of maternal sCD163, maternal IL-6, maternal TNF-alpha and fetal IL-6.

Maternal sCD163	Maternal IL-6		Fetal IL-6		Maternal TNF alpha	
	r	p-value	r	p-value	r	p-value
						
Entire cohort (n = 38)	0.54	0.001*	0.37	0.038*	0.32	0.1
						
GDM (n = 18)	0.61	0.013*	0.71	0.002*	−0.37	0.9
						
Controls (n = 20)	0.16	0.54	−0.25	0.36	0.17	0.56
						

A Pearson product-moment correlation coefficient was computed to assess the relationship between Maternal sCD163 and Maternal IL-6, Fetal IL-6 and Maternal TNF-alpha across the whole cohort, the GDM cohort and the control cohort. (* =  statistically significant.)

* = statistically significant.

### sCD163 level is a predictor for GDM

Together, the above data suggest that chronic inflammation characterizes and correlates with GDM, which is comparable to observations in subjects with T2DM. To determine whether the inflammatory markers were predictors for GDM, we performed stepwise linear regression analysis. We found that maternal IL-6, maternal plasma glucose and maternal age together were significant predictors for GDM status and explained 53% (p<0.0001) of the model, whereas maternal plasma glucose and age alone, without adding IL-6, were poor predictors of GDM status. IL-6 alone or sCD163 alone were significant predictors of GDM status and explained 23% (p = 0.007) and 20% (p = 0.005) of the model, respectively.

### Secretion of sCD163 by paired human placental and adipose tissue explants

**Figure 2:**
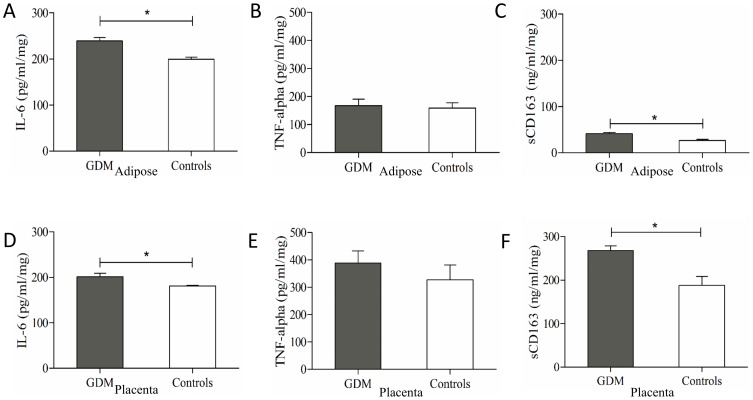
Secretion of IL-6, TNF-alpha and sCD163 from placental and adipose tissue explants. Measurements of adipose (A, B and C) and placental (D, E and F) explants secretion of IL6 (A and D) TNF-alpha (B and E) and sCD163 (C and F). Explants were cultured in CMRL-1066 medium (Invitrogen, Paisley, UK) supplemented with 5% Fetal Calf Serum (Invitrogen, Paisley, UK), 100 µg/ml streptomycin sulphate and 100 IU/ml penicillin. (GDM = gestational diabetes; sCD163 = Soluble CD163; IL-6 = Interleukin 6; TNF-alpha = Tumor necrosis factor alpha [*p<0.05]).

To assess the potential relative contributions of adipose tissue and/or placenta, IL-6, TNF-α and sCD163 were measured in media of paired adipose tissue and placental explants of GDM and non-diabetic mothers. IL-6 levels were significantly elevated in adipose tissue explants from GDM mothers compared to controls (239.08±7.5 vs. 199.43±4.4 pg/ml/mg; p<0.004) (Figure 2A) and similarly, GDM placentae released significantly more IL-6 compared to control placentae (201.26±7.6 vs.180.98±1.1 pg/ml/mg; p<0.02) (Figure 2D). In contrast, adipose tissue-secreted TNF-α levels were not significantly different between GDM (167.28±23 pg/ml/mg) and control mothers (158.86±18.2 pg/ml/mg; p>0.05) (Figure 2B). Likewise, TNF-α secretion from GDM placentae (Figure 2E) was not significantly different compared to control placentae (388.26±44.9 vs.327.52±53.8 pg/ml/mg; p>0.05).

Lastly, adipose tissue explants from GDM mothers released significantly more sCD163 than control (41.06±2.7 vs. 26.56±2.4 ng/ml/mg; p<0.02) (Figure 2C). We observed that human placenta released sCD163; with GDM tissues releasing significantly greater amounts than control placentae (268.07±10.8 vs. 187.55±20.6 ng/ml/mg; p<0.01) (Figure 2F). In contrast to IL-6 and TNF-α, placental explants released significantly more sCD163 than their paired adipose tissue counterparts, suggesting that the placenta might represent a significant source of sCD163.

### CD163 positive cells numbers are increased in placentae of GDM mothers

**Figure 3:**
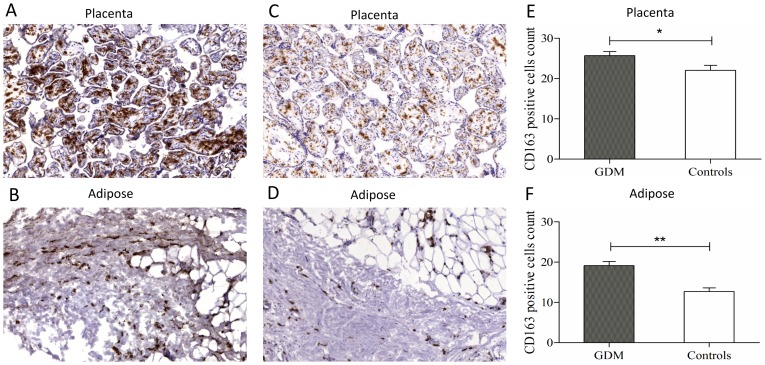
Immunohistochemistry and cell count of CD163 positive cells in GDM (A and B) and controls (C and D) of placental (A and C) and adipose tissues (B and D). CD163 positive cells, in ten random areas at 40× magnification for placenta and adipose tissues respectively were counted and analysed using image J. Mean with SEM of CD163 positive cell in placenta (E) and adipose tissues (F) [*p<0.05, **p<0.001].

Apart from its known role as a general macrophage marker [29], CD163 is also a specific marker of placental macrophages named Hofbauer cells[19]. Given the increased secretion of sCD163 by the placental explants, we compared the numbers of CD163 positive cells in GDM and control placental (Figure 3A & 3C) and adipose tissue (Figure 3B & 3D). We found significantly more CD163 positive cells in GDM placentae than in control placental sections (25.65±1.1vs.22.05±1.2; p<0.03) (Figure 3E). We also identified increased numbers of CD163 positive cells in GDM adipose tissues compared to control adipose tissues (19.1±1.1vs.12.7±0.9; p<0.001) (Figure 3F). We noted significantly more CD163 positive cells in placenta than adipose tissue (p = 0.0004 for GDM and p<0.0001 for control). Together with the increased release of sCD163 in media from placentae, these data suggest that the placenta might represent another potential source of sCD163, in addition to adipose tissue. These data also suggest that Hofbauer cells might be the cellular entity responsible for placental sCD163 secretion.

## Discussion

GDM is an increasingly common metabolic condition in pregnancy, defined by reduced glucose clearance measured during an OGTT [21]. This disorder is not only associated with developing full blown T2DM later in life [5], but also has adverse consequences on the offspring health since the fetus is more likely to develop immediate postnatal hypoglycaemia and has increased lifetime risk to develop obesity and diabetes [6]. An increased inflammatory status in GDM may resemble the low-grade chronic inflammation characterizing T2DM [4]. Here, we demonstrate for the first time elevated circulating maternal levels of the macrophage specific marker sCD163 in GDM, compared to BMI & age matched non-diabetic controls. These findings are in agreement with previous studies on T2DM [11], [30]. We also found, in agreement with previous studies[31] that TNF-α and IL-6 were elevated in GDM pregnancies, which corroborated our findings of elevated sCD163. Although direct assessment of fat mass was not performed, our data likely reflect the consequences or a correlation with GDM and not adiposity since, neither the leptin levels (a biochemical marker of adiposity) nor the gestational weight gain differed between GDM and their age and BMI matched controls. Additionally, in our cohort of overweight and obese women with GDM, co-linearity was shown between sCD163 and maternal IL-6, with increased IL-6 being the driving factor for the observed significant correlation between GDM status and several pro-inflammatory factors. These findings, in part, are at variance with recent findings from others in T2DM [30]. Parkner and colleagues [30] found sCD163, but neither IL-6 nor TNF-α predicting HOMA-IR in multiple linear regression analyses. Although the number of cases was smaller in our cohort, the here observed relation of IL-6 with GDM status might differentiate women with GDM from subjects with T2DM. This assumption is supported by the observation that associations found here were further strengthened when examining the subset of GDM patients, but disappeared in the matched controls. Our results suggest that a combined increase of sCD163 and IL-6 in patients with GDM might differentiate these women from non-pregnant women with T2DM; a finding that requires further investigation.

The placenta has been reported to secrete cytokines and other molecules known to trigger insulin resistance in T2DM[32]. This suggests that the placenta itself might play an important role in the increased inflammation observed in GDM. However, the fact that several placenta-secreted pro-inflammatory molecules are also secreted by adipose tissue complicates defining the role of the placenta in GDM-associated inflammation.

Therefore, in order to attempt to dissect the contributions of placenta and adipose tissue in sCD163 secretion, we investigated paired adipose and placental tissues from GDM and control mothers for their capacity to secrete IL-6, TNF-α and sCD163. We demonstrate for the first time that whilst IL-6 secretion from adipose tissue and placenta was comparable but increased in both tissues in GDM, sCD163 levels from placental explants were significantly higher than those from adipose tissue. In addition, CD163 cell numbers were higher in placenta and adipose tissue of GDM mothers, with placenta containing increased overall numbers of CD163 positive cells compared to adipose tissue. We did not observe statistically significant differences in TNF-α release between GDM and control in either placental or adipose tissue explants, which is in agreement with previous work investigating basal cytokine secretion from placental and adipose tissue explants [31].

These data raised the possibility that the placenta may be an additional source of sCD163 in pregnancy. Intriguingly, CD163 is a known marker of placental macrophages[33] and previous studies have shown higher levels of placental macrophages in obese patients [34]. The increased numbers of CD163 positive cells in placenta of GDM mothers, coupled to our explant data, suggests that placental macrophages may play a role in contributing to circulating sCD163 levels. The release of sCD163 from placental macrophages shows similarity to TNF-α, which is secreted into maternal and fetal circulations by the placenta and is also produced predominantly in placental macrophages[7].

We note that despite a significant increase in Hofbauer cells in GDM placentae (14%), the increases seen in placental explants (≈43%), fetal plasma (≈24%) and maternal plasma (≈38%) are greater than this. Molecular mechanisms and triggering signals leading to sCD163 secretion in GDM remain to be elucidated. We cannot exclude that other inflammatory molecules (e.g. IL-6 from the adipose tissue) stimulated sCD163 production and these molecules might have effects that are both on placenta and adipose tissue. However, according to our regression data, sCD163 alone was a good predictor of GDM; thus, sCD163 may be elevated prior to the diagnosis of GDM, as has already been shown in T2DM [11].

Limitations of our study include the use of estimates of insulin sensitivity rather than measuring this parameter using euglycemic hyperinsulinemic clamps and the lack of information about body fat distribution in the pregnant participants (although leptin was used as a surrogate marker). There were insufficient numbers to determine if there were gender differences in the offspring. Finally, despite the clear evidence of placental secretion of sCD163, the possibility that maternal sCD163 might contribute to the fetal levels, perhaps by crossing the placental barrier, cannot completely be excluded, and further work beyond the scope of this manuscript is required to elucidate this.

In conclusion, we show that sCD163, a known marker of insulin resistance in T2DM, is elevated in GDM. We also show increased numbers of macrophages in both adipose tissue and placentae, with human placenta representing a novel source of sCD163 in pregnancy.
